# Supplementary material for: Mechanism of Morphology Development in HDGEBA/PAMS Hybrid Thermosets: Monte Carlo Simulation and LSCM Study
Source: Polymers (Basel). 2022 Dec 8;14(24):5375. doi: 10.3390/polym14245375 (PMC9788219; doi:10.3390/polym14245375)
Supplement: Supplementary file 1 [file polymers-14-05375-s001.zip › polymers-2035157-supplementary.pdf]

Mechanism of morphology development in hybrid thermosets: Montecarlo simulation and LSCM study

M.G. González, J. Pozuelo, J.C. Cabanelas, B. Serrano, J.Baselga*.

Department of Materials Science and Engineering and Chemical Engineering. University Carlos III of Madrid, 28911 Leganés, Spain

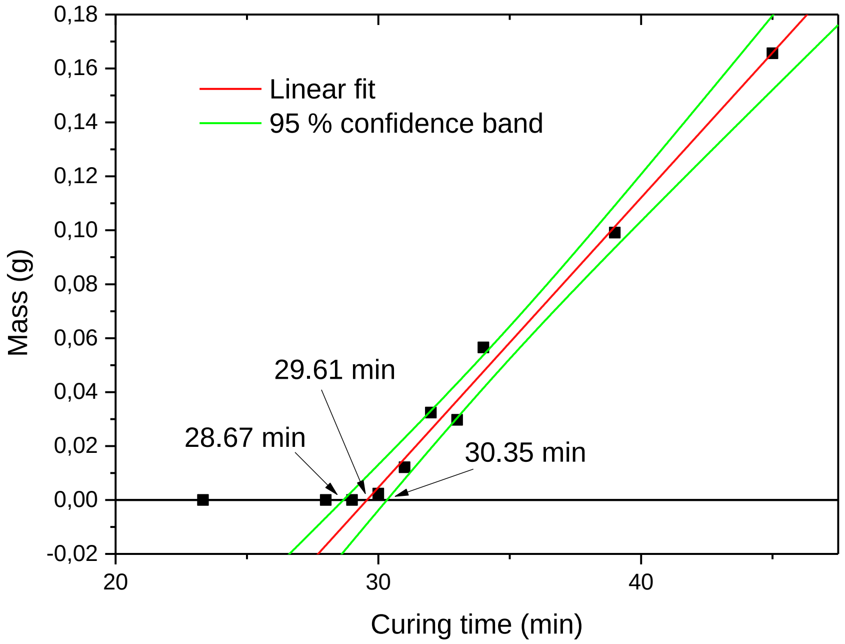

Figure S1. Mass of insoluble fraction of the HFGEBA/PAMS system as a function of curing time. Intercept at Mass = 0 is 29.61 min with an interval of 1.68 min uncertainty at 95% confidence.

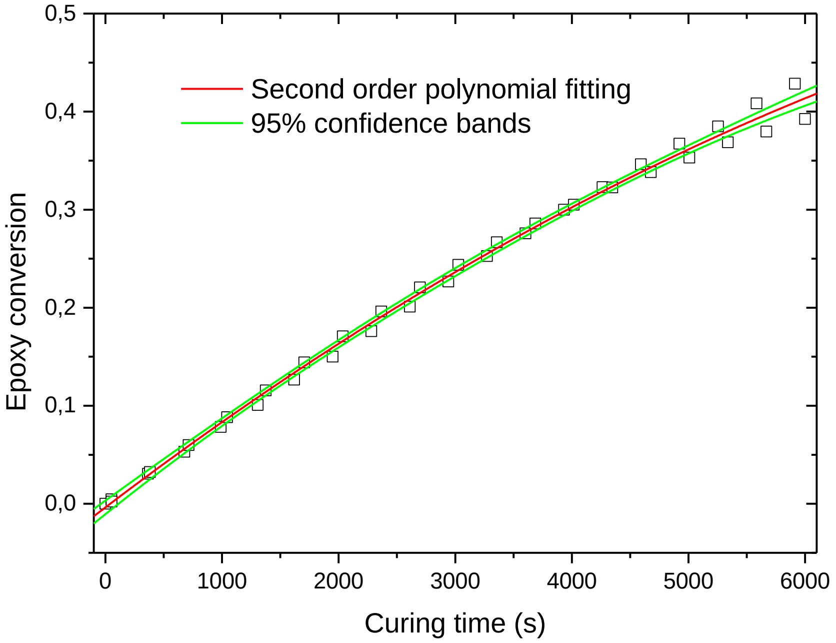

Figure S2. Average time - conversion calibration curves from two experiments at 40 ºC and second order polynomial fit curve along with 95% confidence bands. FTnIR spectra can be found in ref [4] of the manuscript.

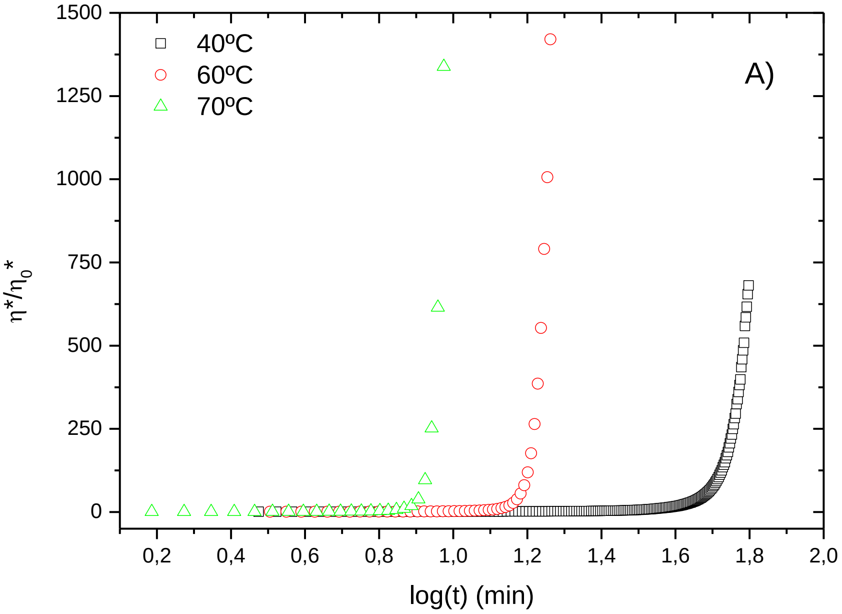

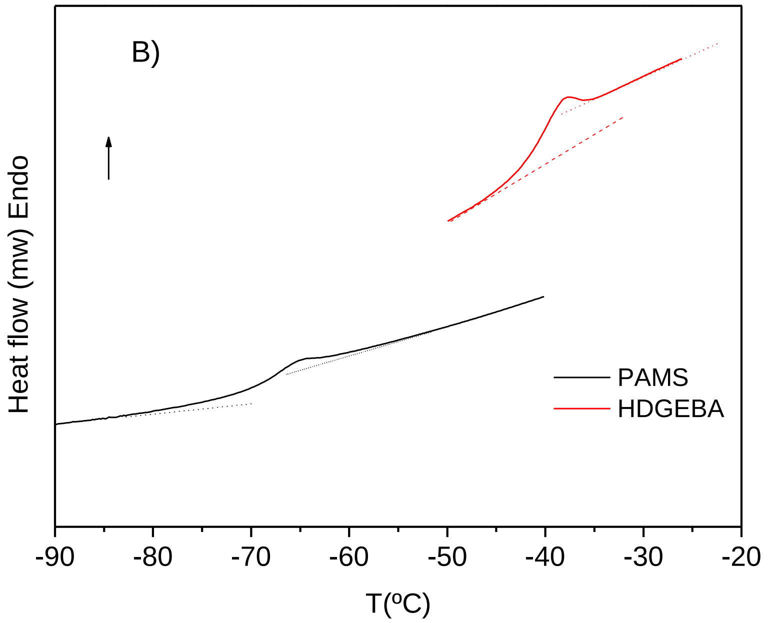

Figure S3. A: Normalized complex viscosity as a function of curing time in log scale for 40ºC, 60ºC y 70ºC curing temperatures. Times at which viscosity diverge are taken as percolation times. B: DSC thermograms of the two pure monomers PAMS and HDGEBA

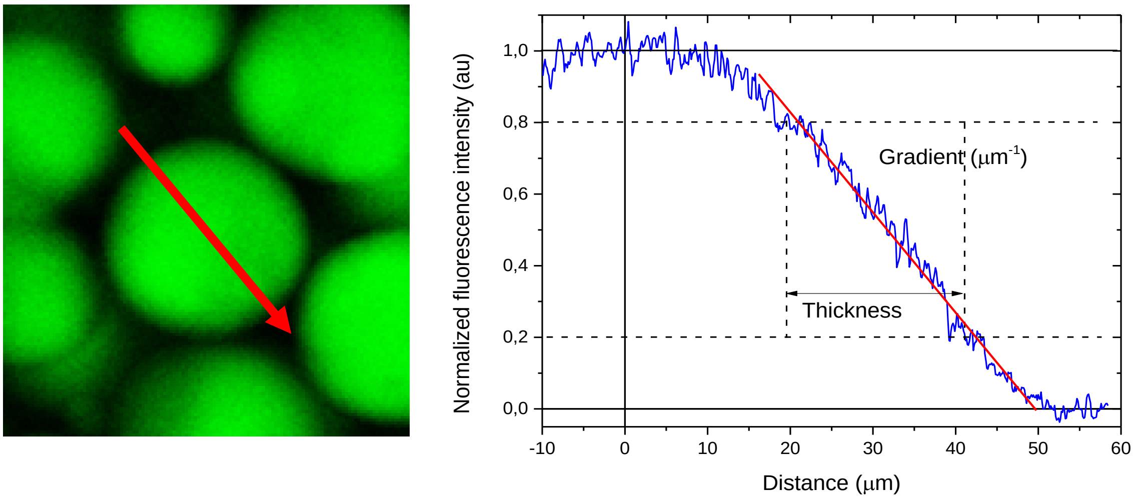

Figure S4. Method to calculate thickness and compositional gradient on a PAMS-rich domain. Interphase thickness is defined as the distance between 20% and 80% of maximum intensity and compositional gradient as the slope between these two limits. The red arrow shows the distance along which fluorescence is recorded.

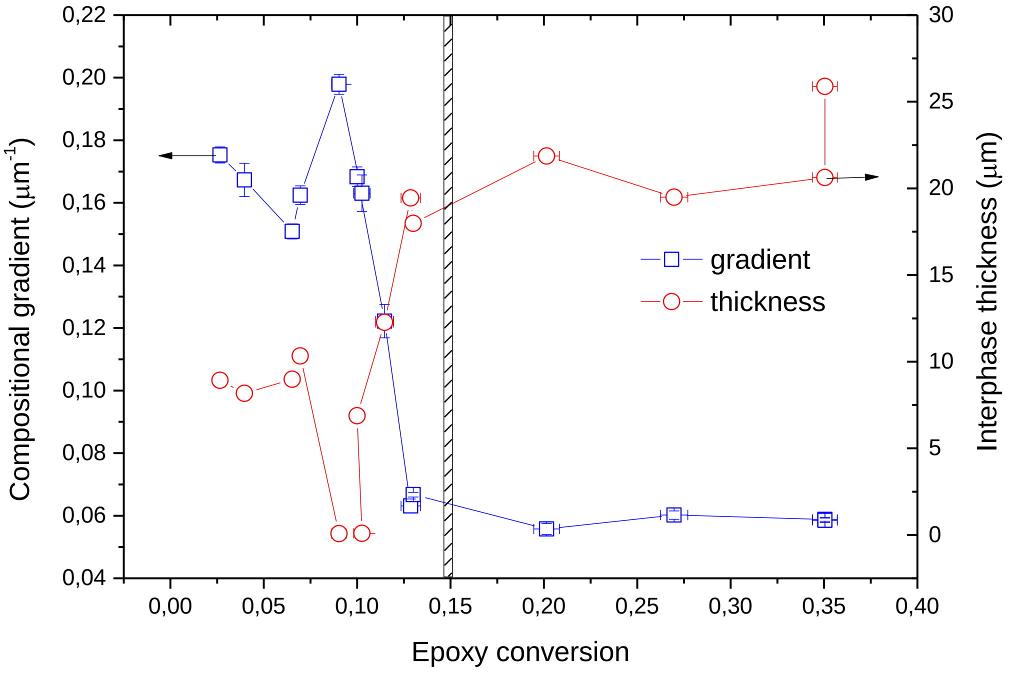

Figure S5. Compositional gradient and interphase thickness as a function of conversion for curing at 40ºC.

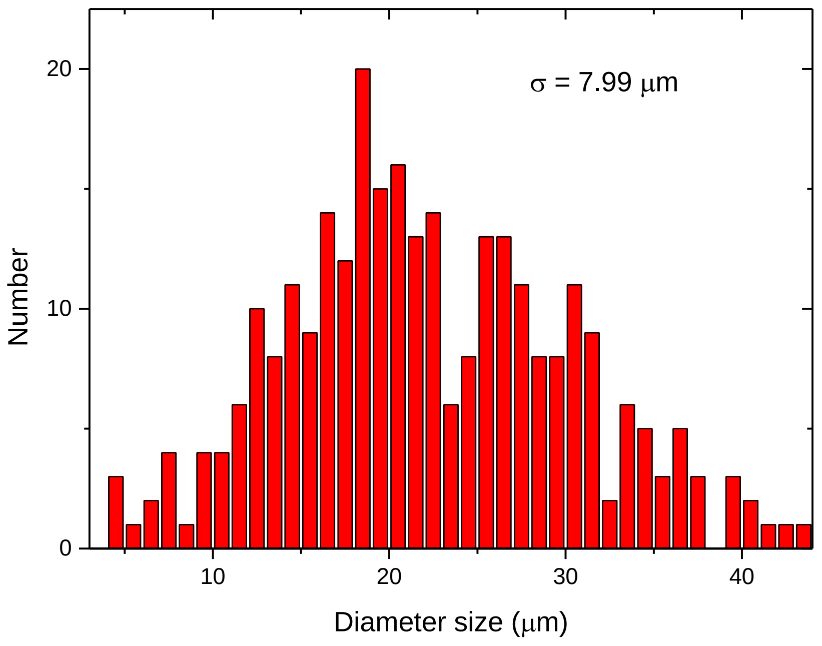

Figure S6. Diameter size distribution at conversion $\alpha = 0$

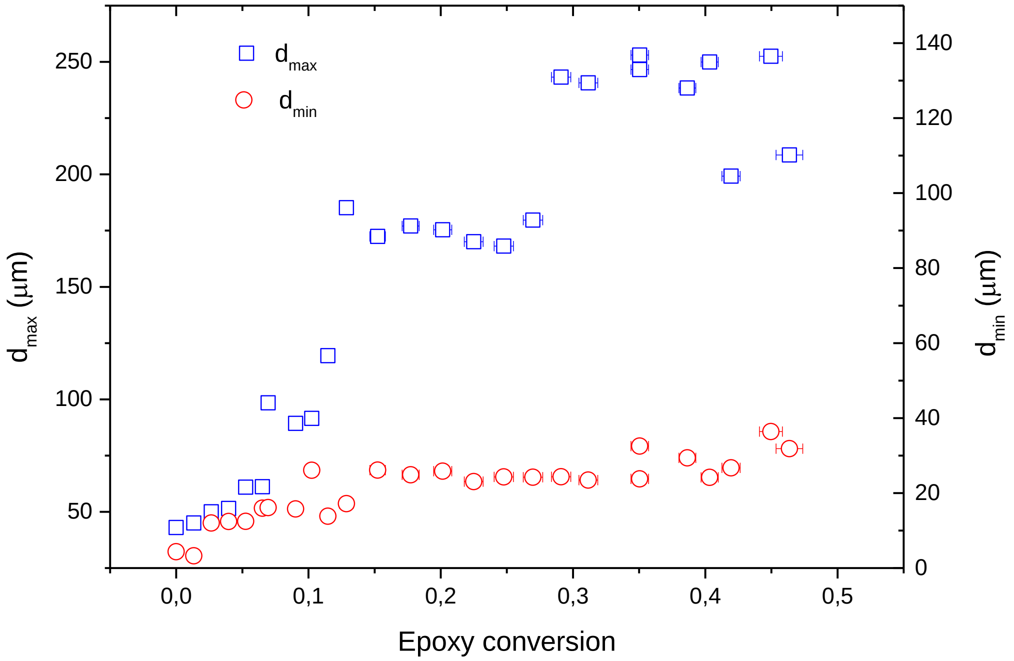

Figure S7. Maximum and minimum droplet diameter as a function of epoxy conversion. Maximum diameter is almost proportional to the third moment of the diameter size distribution.

Appendix: algorithm for the growth and coalescence of domains

Simulation is performed according to the following steps:

1) *Gaussian distribution of particles*.

An initial volume fraction of domains is set, ϕ_D^N, and N particles (~10^5) are generated through Metropolis-Montecarlo with radius in the range $L_R < R_i < U_R$, where L_R and U_R are the lower and upper cut-offs of the distribution. The probability of each size must comply with the Gaussian distribution:

$$y = \frac{1}{\sigma\sqrt{2\pi}} exp\left(-\frac{(R - \langle R\rangle)^2}{2\sigma^2}\right)$$

Where $\langle R\rangle$ is the radius average and σ the radius standard deviation. In the following table appear the initial distributions that have been explored along with the initial volume fractions, average sizes, standard deviations, and lower and upper cut-offs

Run	ϕ_D^N	$\langle R\rangle$ (µm)	σ (µm)	L_R	U_R
1	14	8.0	2.5	2	16
2	16	8.0	2.5	2	16
3	18	8.0	2.5	2	16
4	20	8.0	2.5	2	16
5	25	8.0	2.5	2	16

6	30	8.0	2.5	2	16
7	35	8.0	2.5	2	16
8	40	8.0	2.5	2	16
9	14	10.0	2.5	4	18
10	16	10.0	2.5	4	18
11	18	10.0	2.5	4	18
12	20	10.0	2.5	4	18
13	25	10.0	2.5	4	18
14	30	10.0	2.5	4	18
15	35	10.0	2.5	4	18
16	40	10.0	2.5	4	18
17	14	12.0	2.5	6	20
18	16	12.0	2.5	6	20
19	18	12.0	2.5	6	20
20	20	12.0	2.5	6	20
21	25	12.0	2.5	6	20
22	30	12.0	2.5	6	20
23	35	12.0	2.5	6	20
24	40	12.0	2.5	6	20
25	14	14.0	2.5	8	22
26	16	14.0	2.5	8	22
27	18	14.0	2.5	8	22
28	20	14.0	2.5	8	22
29	25	14.0	2.5	8	22
30	30	14.0	2.5	8	22
31	35	14.0	2.5	8	22
32	40	14.0	2.5	8	22
33	14	16.0	2.5	10	24
34	16	16.0	2.5	10	24
35	18	16.0	2.5	10	24
36	20	16.0	2.5	10	24
37	25	16.0	2.5	10	24
38	30	16.0	2.5	10	24
39	35	16.0	2.5	10	24
40	40	16.0	2.5	10	24
41	14	18.0	2.5	12	26
42	16	18.0	2.5	12	26
43	18	18.0	2.5	12	26
44	20	18.0	2.5	12	26
45	25	18.0	2.5	12	26
46	30	18.0	2.5	12	26
47	35	18.0	2.5	12	26
48	40	18.0	2.5	12	26
49	14	20.0	2.5	14	28
50	16	20.0	2.5	14	28
51	18	20.0	2.5	14	28
52	20	20.0	2.5	14	28
53	25	20.0	2.5	14	28

54	30	20.0	2.5	14	28
55	35	20.0	2.5	14	28
56	40	20.0	2.5	14	28

2) *Stochastic filling of a box*

From the volume occupied by the generated 10^5 particles and the volume fraction value, the volume of the cell is determined. A grid of 75000 was made on each edge of the cell. The filling criterion is as follows: once a particle with radius R is selected, the grid position is randomly selected within the box. Then, it is checked that the grid position is not occupied; if not, a new grid position is randomly chosen, and the process is repeated.

3) *Growth*

The process of growth and coalescence is depicted in the following scheme. Two parent molecules *i,j* grow by a diffusion step of epoxy in which conversion changes from α_k to α_{k+1}. During the diffusion/reaction step, particles remain fixed at their positions.

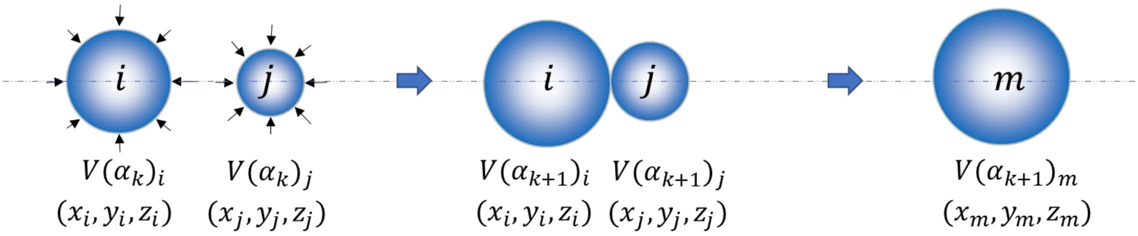

Volume fraction changes with conversion as $\phi(\alpha) = \phi_D^N + \alpha \cdot [1 - \phi_D^N]$ where ϕ_D^N is the initial volume fraction of domains. Therefore, when conversion changes from α_k to α_{k+1}, volume fraction changes as $\Delta\phi(\alpha) = \phi(\alpha_{k+1}) - \phi(\alpha_k) = [1 - \phi_D^N] \cdot [\alpha_{k+1} - \alpha_k]$. Calling $\delta\alpha = [\alpha_{k+1} - \alpha_k]$, the volume of epoxy component that enters the domains is $\Delta V = V_T[1 - \phi_D^N]\delta\alpha$. This volume is distributed among all the particles of the box according to their surface since mass transfer occurs through the surface of domains. Consequently, the volume of each particle, V_i, will increase and amount δV_i given by:

$$\delta V_i = V_T[1 - \phi_D^N]\delta\alpha \cdot \frac{S(\alpha_k)_i}{\sum_{i=1}^{N} S(\alpha_k)_i}$$

The volume of the particle when the system has experienced a conversion change $\delta\alpha$ will now be

$$V(\alpha_{k+1})_i = V(\alpha_k)_i + \delta V_i$$

And the new radius will be

$$R(\alpha_{k+1})_i = \left(\frac{3 \cdot V(\alpha_{k+1})_i}{4\pi}\right)^{\frac{1}{3}}$$

4) *Coalescence*

After each $\delta\alpha$, that has been set at $\delta\alpha = 0.003$, the distance between a particle and the next neighbour $d(\alpha_{k+1})_{ij}$is calculated, considering periodic boundary conditions, as:

- $d_X = X_i - X_j$; $d_Y = Y_i - Y_j$; $d_Z = Z_i - Z_j$

When $d(\alpha_{k+1})_{ij} = \sqrt{d_X^2 + d_Y^2 + d_Z^2} \leq R(\alpha_{k+1})_i + R(\alpha_{k+1})_j$, a coalescent event will occur forming the daughter particle m.

Volume of the new particle will be the sum of the volumes of the parent *i,j* particles and the new radius R_m, will be

$$R_m = \left(R_i^3 + R_j^3\right)^{\frac{1}{3}}$$

The coordinates of the centre will be the coordinates of the centre of mass, given by:

$$X_m = X_i \cdot \frac{R_i^3}{R_i^3 + R_j^3} + X_j \cdot \frac{R_j^3}{R_i^3 + R_j^3}$$
$$Y_m = Y_i \cdot \frac{R_i^3}{R_i^3 + R_j^3} + Y_j \cdot \frac{R_j^3}{R_i^3 + R_j^3}$$
$$Z_m = Z_i \cdot \frac{R_i^3}{R_i^3 + R_j^3} + Z_j \cdot \frac{R_j^3}{R_i^3 + R_j^3}$$
